# Functional principal component analysis as a new methodology for the analysis of the impact of two rehabilitation protocols in functional recovery after stroke

**DOI:** 10.1186/1743-0003-11-134

**Published:** 2014-09-10

**Authors:** M Luz Sánchez-Sánchez, Juan-Manuel Belda-Lois, Silvia Mena-del Horno, Enrique Viosca-Herrero, Beatriz Gisbert-Morant, Celedonia Igual-Camacho, Ignacio Bermejo-Bosch

**Affiliations:** Department of Physical Therapy, University of Valencia, n 5, C/Gascó Oliag, 46010 Valencia Spain; Instituto de Biomecánica de Valencia, Universitat Politécnica de Valencia, Valencia Spain; Grupo de Tecnología Sanitaria del IBV. CIBER de Bioingeniería, Biomateriales y Nanomedicina (CIBER-BBN), 9C 4, Camino de Vera s/n ed, 6022 Valencia Spain; Hospital Universitari i Politècnic La Fe, Bulevar Sur s/n, 46026 Valencia Spain

**Keywords:** Stroke, Functional principal components analysis, Rehabilitation, Physiotherapy, Functional recovery

## Abstract

**Background:**

This study addressed the problem of evaluating the effectiveness of two protocols of physiotherapy for functional recovery after stroke. In particular, the study explored the use of Functional Principal Component Analysis (FPCA), a multivariate data analysis in order to assess and clarify the process of regaining independence after stroke.

**Methods:**

A randomized double-blind controlled trial was performed. Thirteen subjects with residual hemiparesis after a single stroke episode were measured in both in- and outpatient settings at a district hospital. All subjects were able to walk before suffering the stroke and were hemodynamically stable within the first week after stroke. Control and target groups were treated with conventional physiotherapy for stroke, but specific techniques were added for treatment of the target group depending on patients’ functional levels.

Independence level was assessed with the Barthel Index (BI) throughout 7 evolution stages (hemodynamic stability, beginning of standing, beginning of physical therapy sessions in the physiotherapy ward and monthly assessment for 6 months after stroke).

**Results:**

FPCA was applied for data analysis. Statistically significant differences were found in the dynamics of the recovery process between the two physiotherapy protocols. The target group showed a trend of improvement six months after stroke that was not present in the control group.

**Conclusions:**

FPCA is a method which may be used to provide greater insight into the analysis of the rehabilitation process than that provided by conventional parametric methods. So, by using the whole curves as basic data parameters, subtle differences in the rehabilitation process can be found.

FPCA represents a future aid for the fine analysis of similar physiotherapy techniques, when applied in subjects with a huge variability of functional recovery, as in the case of post-stroke patients.

**Electronic supplementary material:**

The online version of this article (doi:10.1186/1743-0003-11-134) contains supplementary material, which is available to authorized users.

## Background

With an ageing population worldwide and the prediction of an ever-increasing burden of stroke-related morbidity and mortality in the elderly, trends of independence recovery have become a key factor in the rehabilitation of subjects who suffer from hemiplegia after stroke [1, 2]. However, there is a huge variability in the functional recovery patterns after stroke [3, 4] and its influence on the statistical analysis of data should be taken into account when predicting the gain associated to a specific physiotherapy protocol as it plays an essential role in rehabilitation research. Moreover, knowing subjects’ recovery dynamics can help to find differences between the evolution of patients treated with different physiotherapy protocols. In this regard, Functional Data Analysis (FDA) offers significant advantages for a better understanding of trends. FDA can extract further information contained in the mathematical function and its derivatives not normally available through traditional statistical methods [5].

In addition, it represents a new conceptualization of the handling of time series and correlated data because this approach treats the whole curve as a single entity. Consequently, there is also no concern about correlations between repeated measurements because it is assumed that an underlying functional relationship governs the data [6, 7].

On the other hand, Principal Component Analysis (PCA) is a multivariate statistical technique, which aims to reduce the dimensionality of high-dimensional data sets by computing another much smaller set of uncorrelated variables (Principal Components), which best represent the original data-set, each new variable being a linear combination of the original ones [5–7]. The main method employed in the statistical analysis of this study was Functional Principal Component Analysis (FPCA). It is based on the PCA however, rather than using variables, the FPCA uses functions [8] to conduct the process. FPCA is one of the most popular multivariate analysis techniques for the extraction of information from FDA.

FPCA is applied for the study of human motion in race walking [6], for the analysis of joint coordination data in motor development [9], for the analysis of the different concurrent mechanisms involved in the learning process [10] and also for forecasting age-specific mortality and fertility rates observed over time [11]. However, its application in studies focused on recovery processes is scarce. FPCA demonstrates how a set of functional data varies with regard to its mean, and in terms of these modes of variability, it quantifies the discrepancy with regard to the mean of each individual functional datum. Accordingly, it could be a useful approach to estimate recovery trends.

The aim of this study was to provide a valid method for the purpose of assessing and clarifying the process of regaining independence after stroke. Particularly, since FPCA is an emerging methodology in the field of rehabilitation research, the objective of this paper was to explore the advantages of this multivariate data analysis over traditional statistical methods in the analysis of functional recovery trends in subjects that presented a huge variability.

## Methods

### Participants

Participants were recruited from Hospital Universitari i Politècnic La Fe of Valencia, Spain. This study was approved by the Hospital’s Ethical Committee and all patients enrolled were informed and they signed informed consent. 13 patients, with a mean age of 73.1 (SD 8.5) were selected based on the following inclusion criteria: having suffered a single stroke episode with residual hemiparesis (regardless of the aetiology and the hemiplegic side), being candidate to begin a rehabilitation programme, being able to walk before suffering the stroke, having the ability to understand and follow simple instructions and being hemodinamically stable within the first week after stroke. Exclusion criteria included poor vital prognosis; pathologies or disorders hampering the development of the study such as: blindness, prosthetics, sensory disorders, severe cognitive impairment and so on; absence of motor impairments after stroke; and pre-stroke disorders that affected the ability to walk.

Participants were randomly assigned to receive either the target or the control treatment; randomization was performed using SPSS 15 for Windows, licensed from the University of Valencia.

### Study design

The study consisted of a randomized double-blind controlled trial: subjects did not know which group they belonged to and the physical therapists making the assessments were blinded to the group treatment assignment. Control and target groups were treated with conventional physiotherapy for stroke, but specific techniques were added to the treatment of the target group depending on patients’ functional levels. Hospital of Sagunto (HS) Functional Scales were used to determine the functional level of each patient [12–14]. Additional techniques used in the target group aimed to strengthen balance, dissociation of movement and sensitivity (Table 1). To avoid differences, the same physiotherapist treated the patients of both groups. Patients were not aware of their group allocation as they were not together at the same time in the physiotherapy unit. All patients underwent five sessions of physiotherapy per week during inpatient rehabilitation and three weekly sessions for outpatient treatment. Each session was 90 minutes long and patients could rest whenever they needed.

**Table 1:** **Description of techniques added to the target group taking into account subjects’ functional level using Hospital of Sagunto Functional Scales**

Functional level	Techniques added to the target group
**BipHS = 0** (Impossible standing)	Plantar stimulation sensitivity*.
**CFMHS = 0** (Nonambulation)	Pelvic dissociation in supine*.
**BipHS = 1** (Nonfunctional standing)	Plantar stimulation sensitivity*.
**CFMHS = 0** (Nonambulation)	Pelvic dissociation in supine*.
	Knee and ankle dissociation in supine**.
**BipHS = 2** (Hand-bound or supported standing)	Plantar stimulation sensitivity*.
**CFMHS = 1** (Nonfunctional ambulation-permanent aid)	Pelvic dissociation in supine*.
	Knee and ankle dissociation in supine**.
	Head dissociations: head rotations in standing with support***.
	Balance: non-affected leg movements, in supported standing***.
**BipHS = 3** (Free, independent and short standing)	Head dissociations: head rotations in standing without support***.
**CFMHS = 2** (Household ambulation-on flat and horizontal surfaces)	
	Balance: non-affected leg movements in standing position***.
**BipHS = 4** (Prolonged standing but abnormal)	Head dissociations: head rotations in standing without support and feet together***.
**CFMHS = 2** (Household ambulation-on flat and horizontal surfaces) or **3** (Surroundings of the house ambulation- restricted distance)	
	Balance: non-affected leg movements in standing position, with feet together***.
**BipHS = 5** (Normal standing)	Head dissociations: head rotations in standing without support, feet together and eyes closed***.
**CFMHS = 3** (Surroundings of the house ambulation- restricted distance) or **4** (Independent community ambulation) or **5** (Normal ambulation- distance, appearance)	
	Balance: non-affected leg movements in standing position, with feet together and eyes closed***.
	Balance: standing in balance board with rotation on horizontal axis and then on vertical axis (when subjects feel confident close their eyes)***.

*Paeth Rohlfs, B. Experiencias con el Concepto Bobath: fundamentos, tratamientos y casos. 2nd Ed. Madrid: Médica Panamericana, 2006, pp 79, 85; **Bobath B. Adult hemiplegia: Evaluation and treatment. 3rd edition. Oxford: Butterworth Heinemann, 1990, p. 100–101;***Adapted from Rose DJ. Equilibrio y movilidad con personas mayores. 1st Ed. Barcelona: Paidotribo, 2005, pp 165–166, 196.

BipHS, Functional Standing Classification of the Hospital of Sagunto (range 0–5); CFMHS, Functional Ambulation Classification of the Hospital of Sagunto (range 0–5).

### Assessments

Assessments were performed by two physical therapists blinded to group treatment assignment. Each physiotherapist assessed subjects independently of the treatment group and the same assessor tested the same participant each time. The inter-assessor reliability was tested and there were no statistical differences, F(1) = 1.96; p = 0.16. The outcome measure was functional ability based on BI which was collected asking the main caregiver. Assessments were performed over a 6-month period after stroke in the following stages: hemodynamic stability, beginning of standing, beginning of physical therapy sessions in the physiotherapy ward and monthly assessment for 6 months after stroke.

### Functional data analysis

The following steps were involved in the functional data analysis of subjects’ recovery dynamics after stroke depending on their physiotherapy treatment: adjusting the curves, calculation of the functional principal components and discriminant analysis. The statistical analysis was made using R [15].

Parametric adjustment of the curves to the equation (1) using the Levenberg-Marquardt algorithm was performed to study the functional recovery curve of patients (based on the BI) and to identify similar recovery patterns. This optimization algorithm was chosen because it allows least squares curve fitting to nonlinear problems such as this case. The mean error of adjustment was below one point on the BI. In the equation, *B(*_*0*_*)* is the initial value of BI when subjects were able to perform the physical therapy session in the physiotherapy unit; *G* refers to the gain, i.e. the final value achieved minus the initial value reached; and the variable *t*_*0*_ indicates the speed at which improvement is achieved.

1 $$B\left(t\right)\;=\;G\;.\;\left(1-\;{e^{–t/t}}^{0}\right)+B_{0}$$

Then functional principal components (FPCs) were calculated and those that explained the percentage of variance were used for the FPCA, following the equation (2), where μ(t) is the mean curve, ξi (t) the functional principal components, (t) the error and *C* i are the scores: a small set of numbers that allows the fitting of each individual curve as a linear combination of the functional principal components.

2 $$B\left(t\right)=\mu \left(t\right)+\sum C_{i}\xi_{i}\left(t\right)+\epsilon \left(t\right)$$

Shapiro-Wilk Test was used to test the normality of the scores. Subsequently, the discriminant analysis was carried out in order to classify each patient within their group and their functional recovery process. Furthermore, the analysis of variance was conducted in order to establish whether the distribution of the two groups was statistically significant. Finally, the mean rate of recovery in each group was analysed to understand the time trend of recovery in both groups.

## Results

Thirteen patients were included in the study, five were randomly allocated to the target group and eight were randomly allocated to the control group. There were no differences between the two groups regarding patient baseline characteristics (Table 2). Group homogeneity was analysed with independent sample *t* test for continuous variables and *χ*^2^ tests for categorical variables.

**Table 2:** **Baseline characteristics of the study subjects (target and control group)**

Patients’ characteristics	Target group (n = 5)	Control group (n = 8)	Baseline comparison (P value)
Age, mean (SD)	70 (7.8)	74.3 (9.4)	0.408
Gender, women, N (%)	2 (40)	5 (62.5)	0.413
Hemorrhages, N (%)	0 (0)	2 (25)	0.359
Affected side, right, N (%)	3 (60)	3 (37.5)	0.413
**Functional state, mean (SD)**			
Barthel Index	13 (10.95)	11.43 (13.13)	0.648
Berg Balance Scale	2.6 (0.89)	6.25 (5)	0.064
CNS	7 (1.15)	7.57 (1.33)	0.565
TCT	46.6 (10.06)	44.63 (20.77)	0.848
FAC	0.2 (0.44)	0.13 (0.35)	0.726

CNS, Canadian Neurological Scale; FAC, Massachusetts General Hospital Functional Ambulation Classification; TCT, Trunk Control Test.

Statistically significant at p < 0.05.

**Figure 1:**
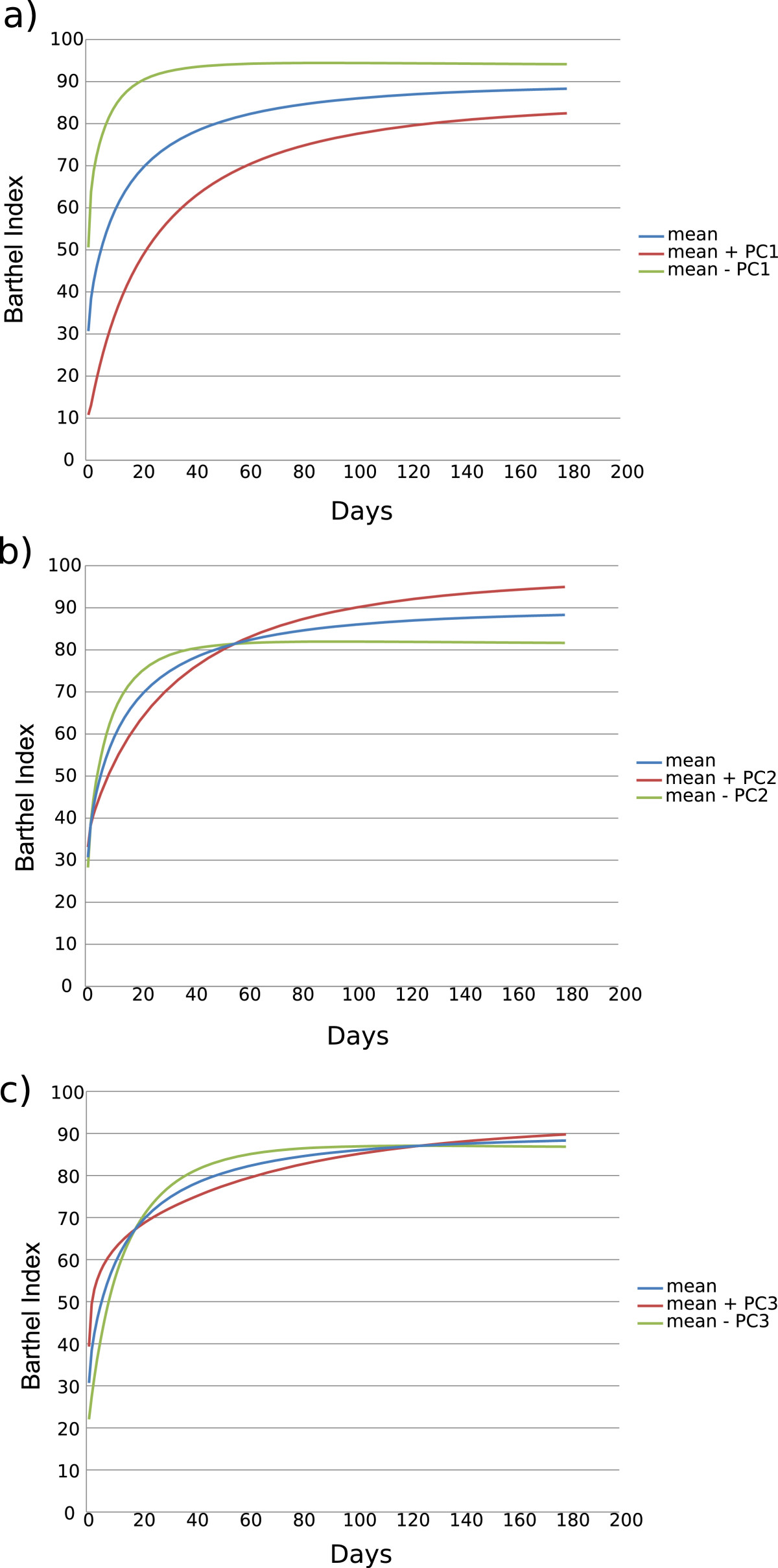
**FPCs for recovery process (BI).** The mean of functional recovery (BI) is shown with curves created by adding (red line) and subtracting (green line) to the mean the standard deviation of scores of FPC1 **(a)**, FPC2 **(b)**, FPC3 **(c)**.

When applying a conventional ANOVA to the data, no differences between both groups (p-Value: 0.331) were found, referred to independence recovery after stroke. On the other hand, the statistical analysis of the data using the previously described FPCA approach gave the following results:

Three FPCs accounted for more than 99% of variance (Table 3). FPCs from the FPCA for functional recovery of all patients over six months after stroke are represented in Figure 1. Analysis of these data showed that FPC1 quantified the general level of independence recovery from the first physiotherapy session in the physiotherapy unit up to six months after stroke. Subjects categorized as having a poorer independence level were found as also having a poorer recovery, while the variability between subjects at the end of the six-month period was lower than at the beginning (Figure 1a). The FPC2 showed certain variability in the functional recovery process among subjects. The level of functional independence was similar in all subjects at the beginning, however, some of them recovered more slowly but reached the highest level of functional independence, while others recovered faster, but reached a lower level of functional independence (Figure 1b). The FPC3 was related to the variability in the middle period of the functional recovery process between subjects (Figure 1c).

**Table 3:** **Percentage of variance explained by the model**

	FPC1	FPC2	FPC3
Standard deviation	11.19	5.01	2.12
Proportion of Variance	80%	16%	3%
Cumulative Proportion	80%	96%	99%

**Figure 2:**
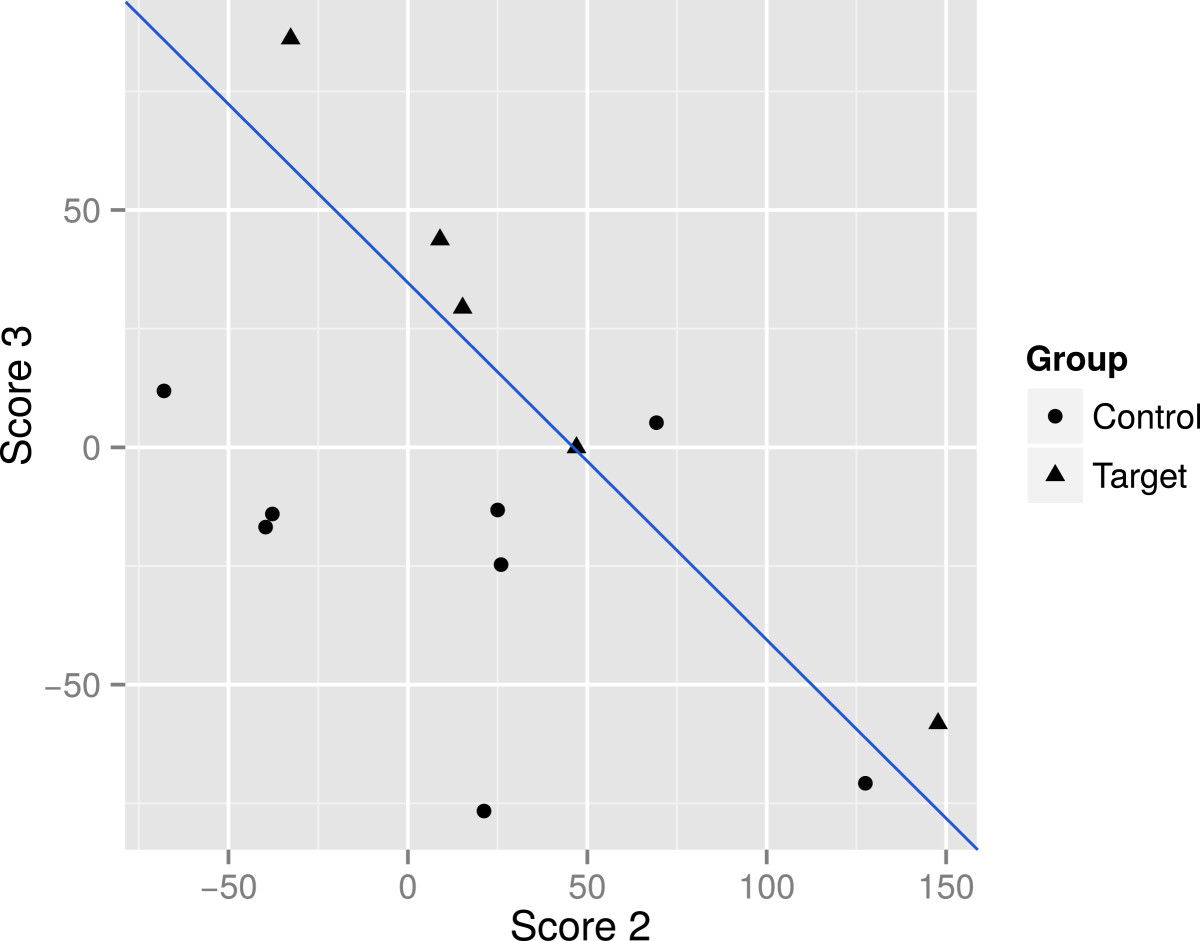
**Discriminant analysis to classify subjects in groups of functional recovery.** The figure shows the scatterplot of score 2 versus score 3. The axes X and Y are the scores (*C* i) obtained according to equation (2). Line shows graphically that subjects in the treatment group are above the line, except one, meanwhile, subjects in the control group are below the line.

Normality assumption was tested with Shapiro*-* Wilk test and normality was assumed in all the scores (p-value higher than 0.05). The p-value for score 1 was 0.059, for score 2 it was 0.760 and p-value for score 3 was 0.940. Moreover, the linear discriminant analysis showed that both groups could be classified according to the FPCs (Figure 2). Finally, the analysis of variance between groups was statistically significant (p-value 0.005).

**Figure 3:**
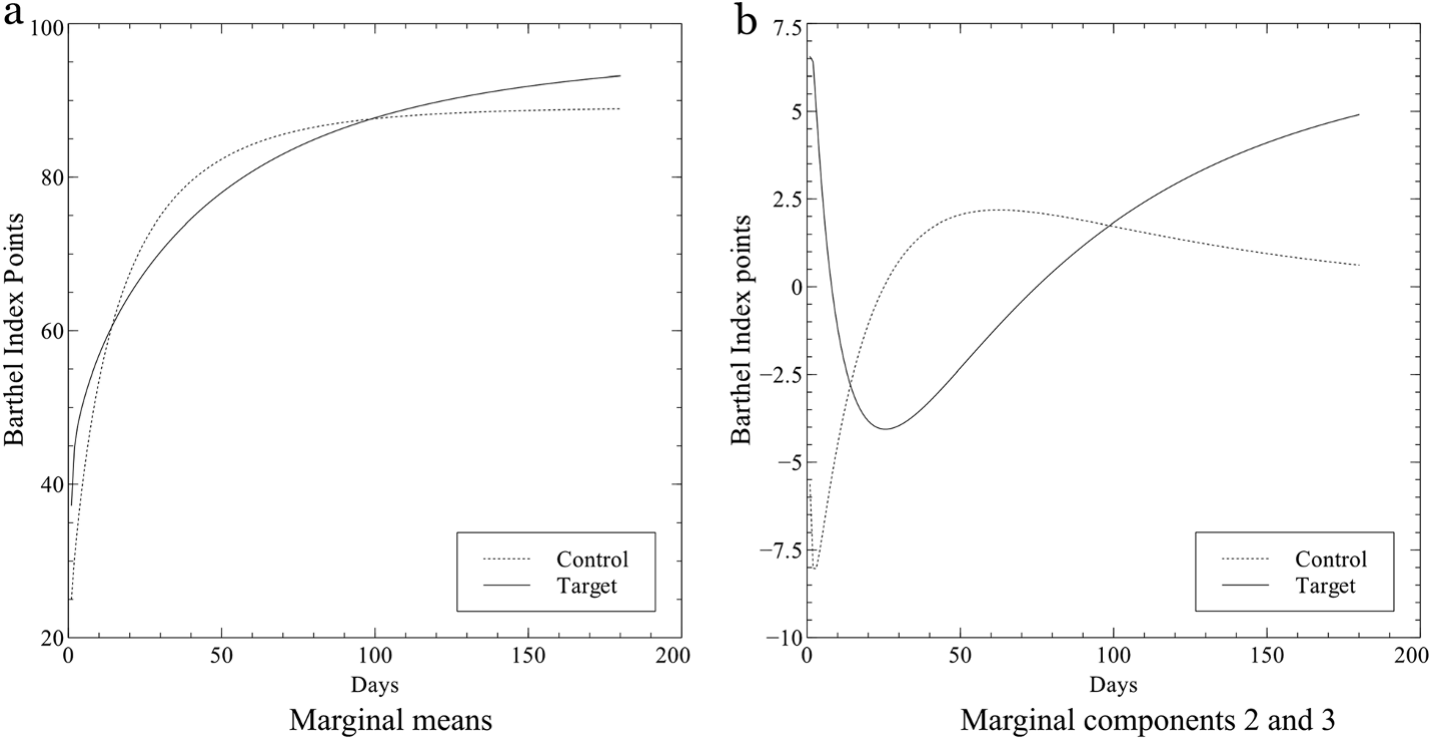
**Differences in the recovery dynamics from FPC2 and FPC3.** Differences in the recovery dynamics between groups can be observed in this figure. Reconstructed curves from the marginal mean components per group obtained in the FPCA showed that target group has a trend of improvement after six months of stroke while control group did not have it **(a)**. Differences in dynamics are more evident when just the functional principal components 2 and 3 are reconstructed **(b)**.

In order to interpret the dynamics of recovery in each group, the marginal mean curves (Figure 3) were reconstructed from the values of the means per group (Table 4). Accordingly, Figure 3 shows the trends in functional recovery (BI) for both groups (target and control) over the six-month period after stroke. Functional recovery increased with time in both groups, however, the target group continued to improve after this period while the control group trend remained constant.

**Table 4:** **The table shows the means per group from which has been reconstructed the marginal mean curves of both groups**

Means per group		
	Score 2	Score 3
Control Group	15.4	−24.9
Target Group	37.2	20.2

## Discussion

For the analysis of function recovery process after stroke, particular attention must be paid to the huge inter-subject variability. Probably due to this factor, various studies [16, 17] in this research area have not found statistically significant differences through the use of standard statistical analysis techniques. In this paper, traditional analysis (ANOVA) and FPCA were applied to study the functional recovery of 13 subjects who randomly participated in two physiotherapy protocols in order to establish which was more suitable for function recovery after stroke. The ANOVA between groups was not significant (p-value 0.331), however, we found statistically significant differences through FPCA as this method deals with the subjects’ dynamics of recovery instead of particular values at given times. Therefore, through the use of FPCA the relevance of additional techniques used in the target group for the functional recovery process after stroke was established. Donà et al. [6] also studied the limitations of conventional statistical analysis and they demonstrated the potential benefits of FDA, providing greater insight into subtle differences in kinematic and kinetic patterns for athletes having different skill levels. However, they did not study the effect of any therapy and to our knowledge, there are scarce studies using FPCA to analyse the results of different rehabilitation treatments for functional recovery after stroke [5]. Thus, FPCA is a useful approach to estimate recovery trends when variability between subjects is very large, as accounting for inter-subject variability ensures that essential trends for recovery patterns are not overlooked due to limitations of the statistical analysis procedures [6].

It is widely known that recovery after stroke usually occurs in the first six months following the event [18, 19]. However, we found a significant difference in the dynamics of recovery between groups. A positive trend of recovery which continued after 6 months following the stroke was found in the target group, while a plateau was observed in the recovery process of the control group. Moreover, we can be sure that these differences were due to the physiotherapy protocol because both groups were homogeneous at baseline characteristics and there were no differences in the FPC1 which quantifies the general level of independence recovery from the first session in the physiotherapy ward up to six months after stroke. It showed that subjects categorized with a poorer level, likewise had a poorer recovery, which agrees with the findings of other authors in the literature [20]. Additionally, independence recovery was faster during the first three months after stroke as suggested by other authors [18, 21–24]. Moreover, FPC2 refers to a trend of variability based on the differences in the time of recovery; the results showed that it was statistically significant for the treatment group, p-value less than 0.05 (0.005). Finally, FPC3, that refers to recovery trend development in the middle period of recovery, was statistically significant during inpatient rehabilitation (p-value 0.014), which means that subjects had a lower level of independence.

Therefore, this methodology is able to clarify the process of functional recovery after stroke. So, as first component of this pattern accounts for 80% of total variance, the final functional outcome mainly depends on subject’s initial condition: individuals with lower BI rates at the beginning of the recovery reach lower BI rates six months after stroke and recover more slowly than subjects with higher BI at the beginning (Figure 1a). Moreover, 16% of variance is accounted for the physiotherapy protocol applied, suggesting that the use of specific physiotherapy techniques can influence the recovery process and final outcomes even beyond six months after stroke.

Finally, determining if a protocol of physiotherapy is more appropriate than other is a hard task as researchers find several limitations. Mainly, the size of patients is often small [25] because it is very difficult to find an adequate amount of subjects who meet the inclusion criteria [1, 12, 21] and frequently, there is a huge variability in functional recovery process between subjects after stroke [3, 4]. These limitations were also found in the present study.

Nevertheless, FPCA has proven to be useful for the detection of significant outcomes that are not found by the use of conventional statistical analysis techniques. The main limitation of FPCA is the difficulty to interpret the results. Interpretation of results in parameter based analysis is straightforward, however, in FDA and particularly in FPCA, interpretation is based on the whole function.

In short, FPCA has some limitations but it is useful to analyse recovery trends when variability is very large while improvements do not so much involve increased scores on a given scale (i.e. BI), yet relate to the dynamics of recovery. Thus, in this paper we used FPCA as a method to assess differences in the recovery processes of subjects after stroke, thereby establishing which techniques are more effective than others and promoting standardized treatment protocols in the rehabilitation area. Future research for the application of FPCA in larger samples could be interesting specially for determining which physical therapy techniques are more effective.

## Conclusions

FPCA provides a useful approach for the purpose of analysing recovery trends because it deals with subjects’ dynamics of recovery and not with specific values at given times. Furthermore, FPCA allows the detection of statistically significant differences hardly detectable through conventional statistical analysis. So this methodology could be useful for the determination of functional recovery patterns after stroke.

This type of analysis can be used to establish differences between treatments when subjects present a huge variability, this being one of the largest research gaps in the field of stroke rehabilitation. In fact, further study on the influence of different physiotherapy protocols on functional recovery after stroke is needed in order to determine which techniques are more effective.

Finally, study results support the use of FPCA in stroke rehabilitation and other research in health sciences where initial measurements involve time series data and huge variability.

## Authors’ original submitted files for images

Below are the links to the authors’ original submitted files for images. Authors’ original file for figure 1 Authors’ original file for figure 2 Authors’ original file for figure 3
