# Supplementary figures and images for: Functional principal component analysis as a new methodology for the analysis of the impact of two rehabilitation protocols in functional recovery after stroke
Source: J Neuroeng Rehabil. 2014 Sep 10;11:134. doi: 10.1186/1743-0003-11-134 (PMC4246446; doi:10.1186/1743-0003-11-134)

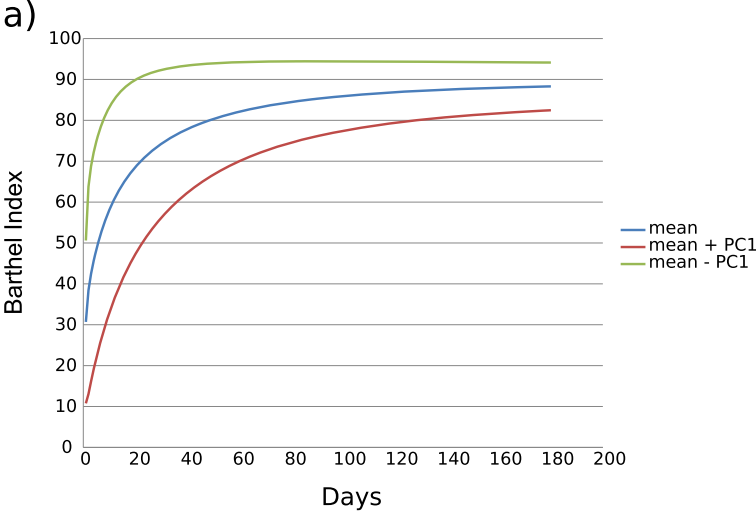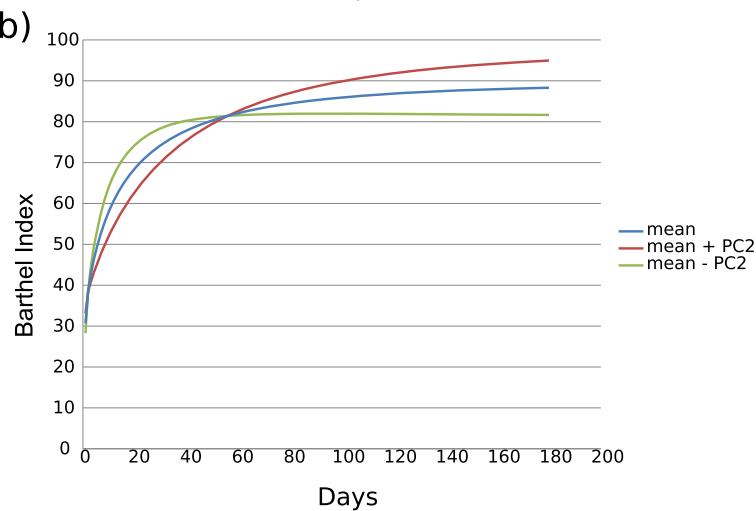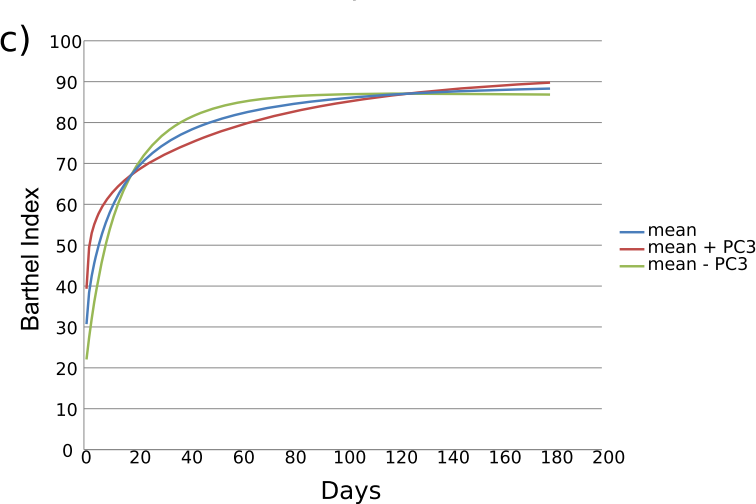

Supplement: Supplementary file 1 — Authors’ original file for figure 1 [file 12984_2014_673_MOESM1_ESM.pdf]

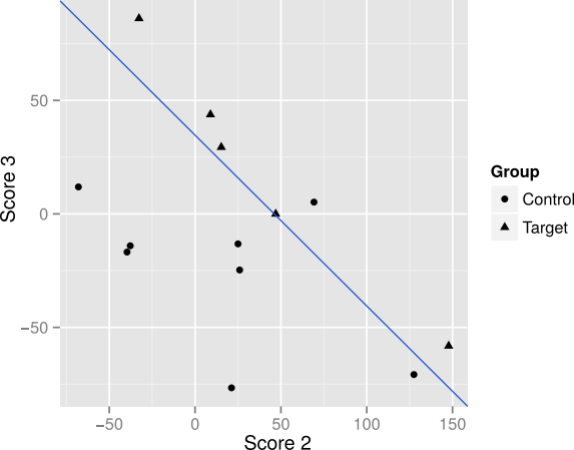

Supplement: Supplementary file 2 — Authors’ original file for figure 2 [file 12984_2014_673_MOESM2_ESM.pdf]

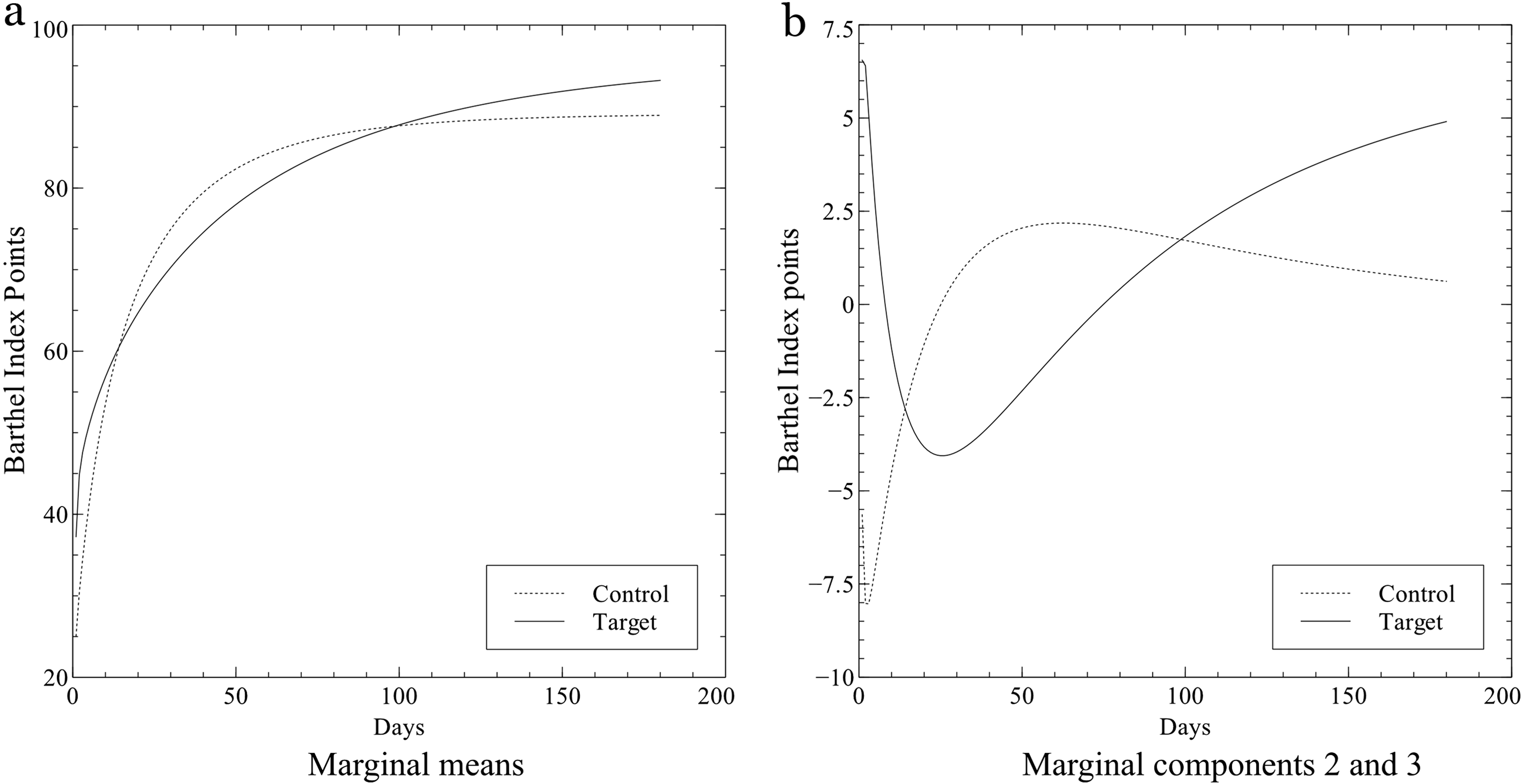

Supplement: Supplementary file 3 — Authors’ original file for figure 3 [file 12984_2014_673_MOESM3_ESM.tiff]
